# Activation, but not inhibition, of the indirect pathway disrupts choice rejection in a freely moving, multiple-choice foraging task

**DOI:** 10.1016/j.celrep.2022.111129

**Published:** 2022-07-26

**Authors:** Kristen Delevich, Benjamin Hoshal, Lexi Z. Zhou, Yuting Zhang, Satya Vedula, Wan Chen Lin, Juliana Chase, Anne G.E. Collins, Linda Wilbrecht

**Affiliations:** 1 Department of Psychology, University of California, Berkeley, Berkeley, CA 94720, USA; 2 Helen Wills Neuroscience Institute, University of California, Berkeley, Berkeley, CA 94720, USA; 3 Department of Molecular and Cell Biology, University of California, Berkeley, Berkeley, CA 94720, USA; 4 Department of Integrative Physiology and Neuroscience, College of Veterinary Medicine, Washington State University, Pullman, WA 99164, USA; 5 Lead contact

## Abstract

The dorsomedial striatum (DMS) plays a key role in action selection, but less is known about how direct and indirect pathway spiny projection neurons (dSPNs and iSPNs, respectively) contribute to choice rejection in freely moving animals. Here, we use pathway-specific chemogenetic manipulation during a serial choice foraging task to test the role of dSPNs and iSPNs in learned choice rejection. We find that chemogenetic activation, but not inhibition, of iSPNs disrupts rejection of nonrewarded choices, contrary to predictions of a simple “select/suppress” heuristic. Our findings suggest that iSPNs’ role in stopping and freezing does not extend in a simple fashion to choice rejection in an ethological, freely moving context. These data may provide insights critical for the successful design of interventions for addiction or other conditions in which it is desirable to strengthen choice rejection.

## In brief

To test the role of indirect and direct pathway spiny projection neurons (SPNs) in active choice rejection, Delevich et al. ran mice in a serial choice task with chemogenetic manipulation. Activation, but not inhibition, of iSPNs within the dorsomedial striatum (DMS) impaired active choice rejection, contrary to heuristic predictions.

## Graphical abstract

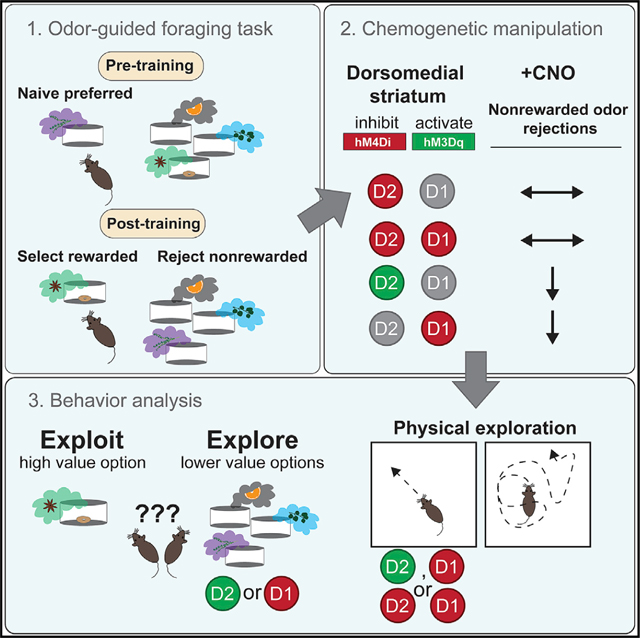

## INTRODUCTION

In everyday decision making, we often consider multiple options in a serial fashion, foregoing low-value choices to ultimately arrive at a higher-value choice. As time passes and the environment or our needs change, we also learn to reject formerly high-value choices to adjust our behavior to new contingencies or states. The inability to reject problematic choices is a key component of addiction, eating disorders, and obsessive-compulsive disorder. The mechanisms underlying learned choice rejection are therefore highly relevant to psychiatry and public health.

The dorsomedial striatum (DMS; homologous to the primate caudate) is a key brain structure for goal-directed action selection (Balleine and O’Doherty, 2010), and striatal dysfunction is associated with maladaptive choice behavior (Everitt et al., 2008; Foerde et al., 2015; Friedman et al., 2017). However, it is still not well understood how value-based choice, and choice rejection in particular, are implemented at the circuit level (Cox and Witten, 2019). Furthermore, much of the relevant functional data comes from two-alternative forced choice (2AFC) tasks (Donahue et al., 2018; Kim et al., 2009; Tai et al., 2012; Kwak and Jung, 2019; Lee and Sabatini, 2021), in which movement is constrained and it is difficult to dissociate the selection of one choice (e.g., turn left) from the rejection of another (e.g., do not turn right). Therefore, studying DMS function in a task in which animals move freely and select among multiple options may reveal new insights into the circuit mechanisms that underlie choice selection and active rejection.

The majority of neurons in the DMS are spiny projection neurons (SPNs) whose activity reflects task features including movement, cues, and value (Isomura et al., 2013; Nonomura et al., 2018; Shin et al., 2018; Thorn et al., 2010). Within the DMS, SPNs with different projections express different dopamine receptors, with direct pathway SPNs (dSPNs) expressing dopamine D1 receptors and indirect pathway SPNs (iSPNs) expressing dopamine D2 receptors (Gerfen et al., 1990). Consistent with predictions from functional neuroanatomy (Alexander and Crutcher, 1990; Mink, 1996) and theoretical work (Collins and Frank, 2014; Frank et al., 2004), optogenetic stimulation of dSPNs promotes movement and reinforces actions (“go” functions), whereas optogenetic stimulation of iSPNs inhibits movement and drives aversion (“no go” functions) (Kravitz et al., 2010, 2012; Yttri and Dudman, 2016; Tecuapetla et al., 2016). In a 2AFC task, DMS dSPN stimulation promotes contraversive choices whereas DMS iSPN stimulation promotes ipsiversive choices in a manner that is reward-history dependent (Tai et al., 2012). These effects are also observed in freely moving contexts (Tecuapetla et al., 2014) and have recently been replicated in a head-fixed lateralized licking task when stimulation is focused on the ventrolateral striatum (Lee and Sabatini, 2021).

**Figure 1.**
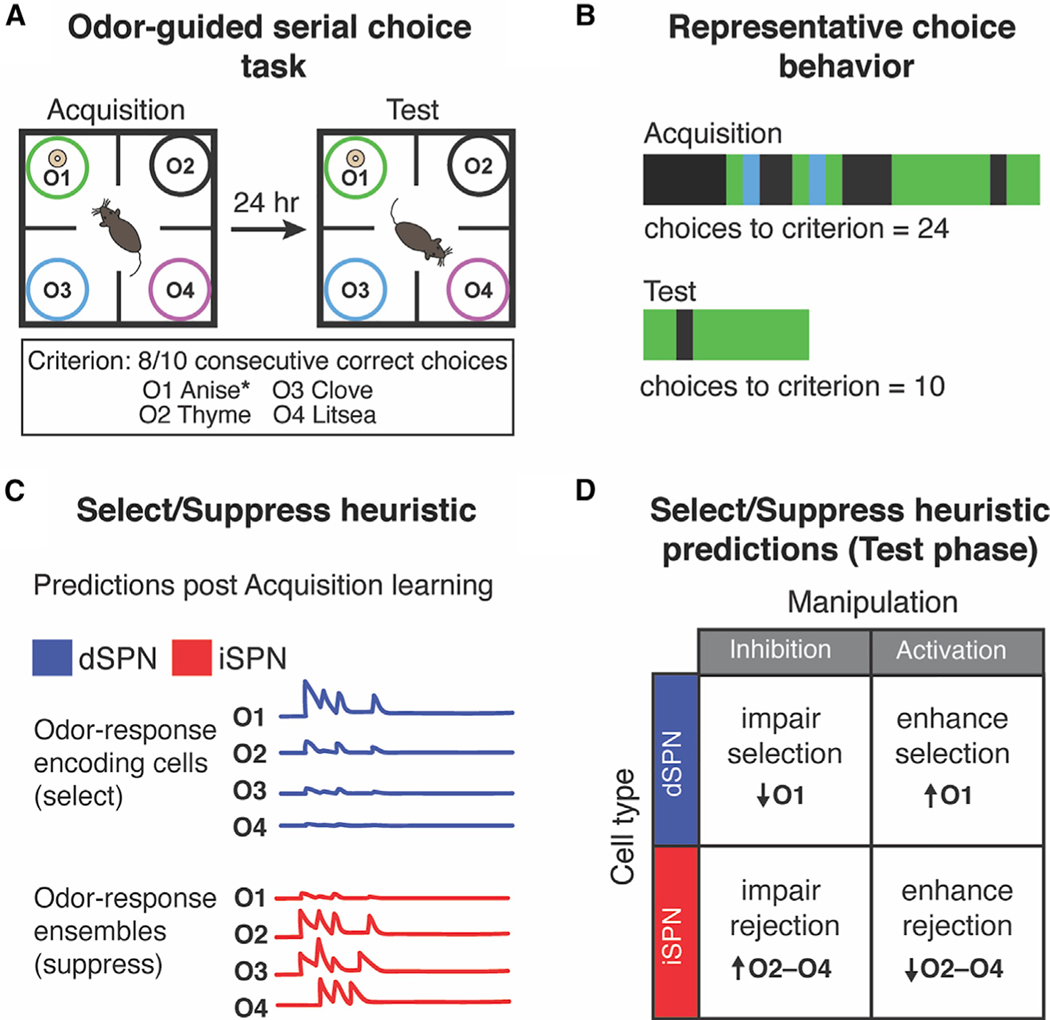
Select/suppress heuristic predictions for SPN manipulation in odor-guided serial choice task (A) Task schematic. (B) Odor choices made during acquisition (top) and test (bottom) phases from representative mouse. Vertical bars indicate odor choice on single trial. (C) Putative activity patterns of dSPN and iSPN ensembles for each odor choice, illustrating basic assumptions of simple select/suppress heuristic model. (D) Select/suppress heuristic predictions for dSPN and iSPN manipulation during test phase. (C) is based on Extended Data Figure 10 in Parker et al., (2018).

While these data suggest dichotomous function of striatal dSPNs and iSPNs in decision-making, *in vivo* recordings have shown that they are co-active during goal-directed and spontaneous movement (Cui et al., 2013; Isomura et al., 2013; Jin et al., 2014; Tecuapetla et al., 2014; Parker et al., 2018; Markowitz et al., 2018) and that balanced co-activation is important for movement selection (Tecuapetla et al., 2014; Parker et al., 2018). To reconcile these observations, it has been proposed that the two pathways work in concert such that dSPN activity promotes desired actions/choices while iSPN activity suppresses competing actions/choices (Cui et al., 2013; Hikosaka et al., 2000; Mink, 1996). In its simplest form, this select/suppress heuristic model assumes that increases in dSPN activity are important for choice selection whereas increases in iSPN activity are important for choice suppression or rejection (Figure 1). Here, we sought to test this heuristic in the context of serial choice and, in particular, determine how bidirectional manipulation of iSPNs affects learned choice rejection.

## RESULTS

### Select/suppress heuristic predicts iSPN inhibition will impair learned choice rejection in an odor-guided serial choice task

We trained mice in an odor-guided task in which they approach distinctly scented pots in a serial fashion, rejecting pots until they choose one by digging in the scented shavings it contains (Johnson et al., 2016) (Figure 1A). Only one of four odors was rewarded (O1, anise), and mice learned the odor-action-reward contingency through trial and error during an acquisition phase. Training-naive mice exhibited a consistent preference for a particular nonrewarded odor (O2, thyme; see STAR Methods). Therefore, acquisition involved both learning to select O1 and to reject digging to O2, the recall of which was assessed during a test phase the following day (Figures 1A and 1B; STAR Methods).

If iSPN activity is responsible for choice rejection as suggested by a simple version of the select/suppress heuristic (Figure 1C), we reasoned that inhibiting iSPNs during the test phase should lead to more choices to O2–O4 (Figure 1D). Conversely, activation of iSPNs should enhance rejection of nonrewarded choices or, alternatively, result in choice omission due to rejection of all choices (Figure 1D). For the dSPNs, we predicted that chemogenetic inhibition would impair selection of O1 whereas activation would enhance selection of O1 (Figure 1D).

### Chemogenetic activation, not inhibition, of the indirect pathway impairs the ability to reject nonrewarded choices

**Figure 2.**
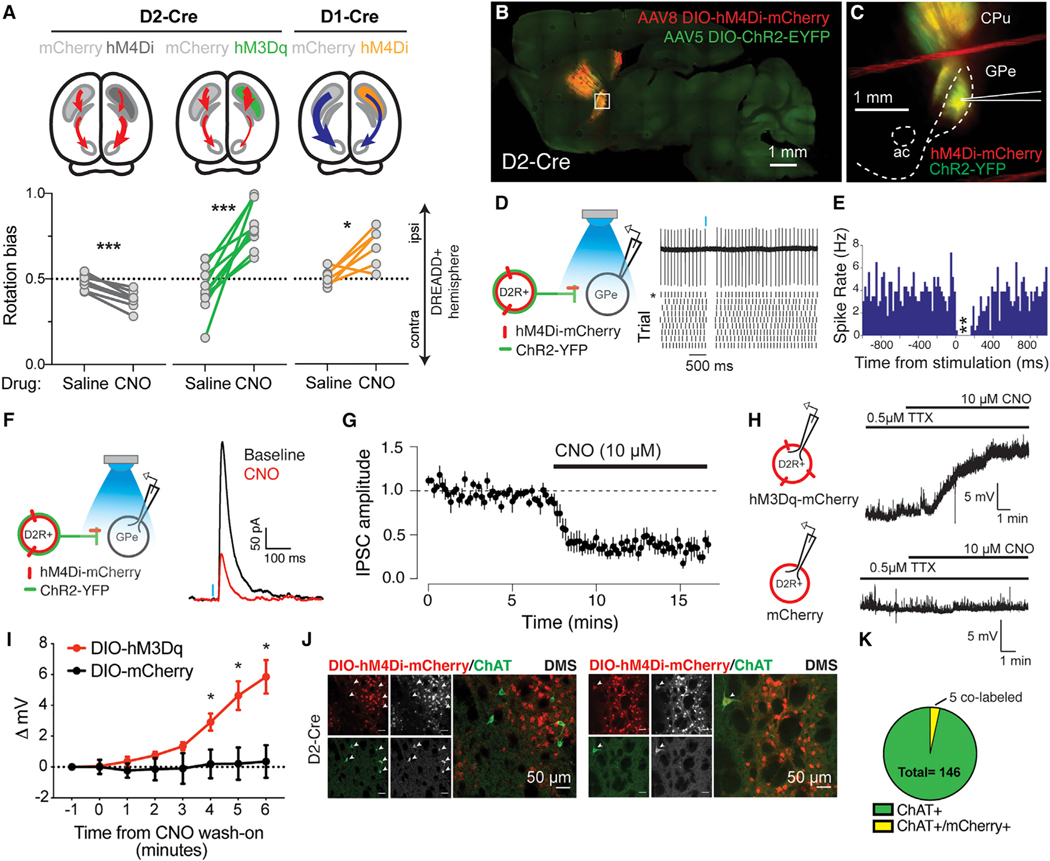
Establishing efficacy of DREADDS in DMS iSPNs and dSPNs (A) Unilateral chemogenetic inhibition of dSPNs versus iSPNs had opposite effects on rotation bias: dSPN inhibition drove a significant ipsiversive bias (*p = 0.02), and iSPN inhibition drove a significant contraversive bias (***p < 0.0001). Unilateral chemogenetic activation of iSPNs drove a significant ipsiversive bias (***p < 0.0001) (n sessions/N mice = 8/4, 10/5, and 6/3). (B) D2-Cre mice were co-transduced with Cre-dependent hM4Di-mCherry and Cre-dependent ChR2-EYFP into DMS. Scale bar: 1 mm. (C) Sagittal slice containing globus pallidus externa (GPe) targeted for patch-clamp recording. (D) Cell-attached recording configuration. Asterisk indicates raster for raw trace above. (E) Peristimulus spike histogram; blue light stimulus significantly reduced spike rate (n cells/N mice = 6/3). (F) Whole-cell recording configuration: sample evoked inhibitory postsynaptic current (eIPSC) before and after CNO (10 μM) wash on. (G) Normalized eIPSC amplitude before and after CNO wash on (***p < 0.0001) (n cells/N mice = 6/3). (H) mCherry+ cells were targeted for whole-cell current clamp recording in D2-Cre mice transduced with Cre-dependent hM3Dq-mCherry or mCherry virus. CNO (10 μM) was bath applied in the presence of TTX (0.5 μM). (I) CNO depolarized hM3Dq-mCherry+, but not mCherry+, cells. (***p < 0.0001) (n cells/N mice = 5/3, 6/4). (J) Top panel: representative images show lack of co-localization of Cre-dependent mCherry (red) and choline acetyltransferase (ChAT) immunoreactivity (green) within DMS of AAV8-hSyn-DIO-mCherry-transduced D2-Cre mice (N = 4). White arrows indicate ChAT+ neurons. Bottom panel: rare co-localization of Cre-dependent mCherry (red) and ChAT (green). White arrow indicates ChAT+/mCherry+ neuron. (K) Proportion of ChAT+/mCherry+ neurons within regions of AAV8-hSyn-DIO-mCherry infection. Mean ± SEM shown in (G) and (I). See full statistics in Table S2.

To test these heuristic predictions, we performed *in vivo* chemogenetic manipulation using designer receptors exclusively activated by designer drugs (DREADDs). Efficacy of activating (hM3Dq) and inhibitory (hM4Di) DREADDs was confirmed via *in vivo* rotational studies with unilateral manipulations (Figure 2A) and *ex vivo* slice electrophysiology experiments (Figures 2B–2G). In slice, CNO activation of hM4Di suppressed iSPN synaptic release (Figures 2F and 2G) and CNO activation of hM3Dq depolarized iSPNs (Figures 2H and 2I). Histological examination revealed a low incidence of mCherry and choline acetytransferase (ChAT) co-labeling in D2-Cre mice (Figures 2J and 2K), similar to what was previously reported between Adora2A-Cre expression and ChAT in dorsal striatum (Lemos et al., 2016). These results indicate that infection was largely restricted to iSPNs in D2-Cre mice.

**Figure 3.**
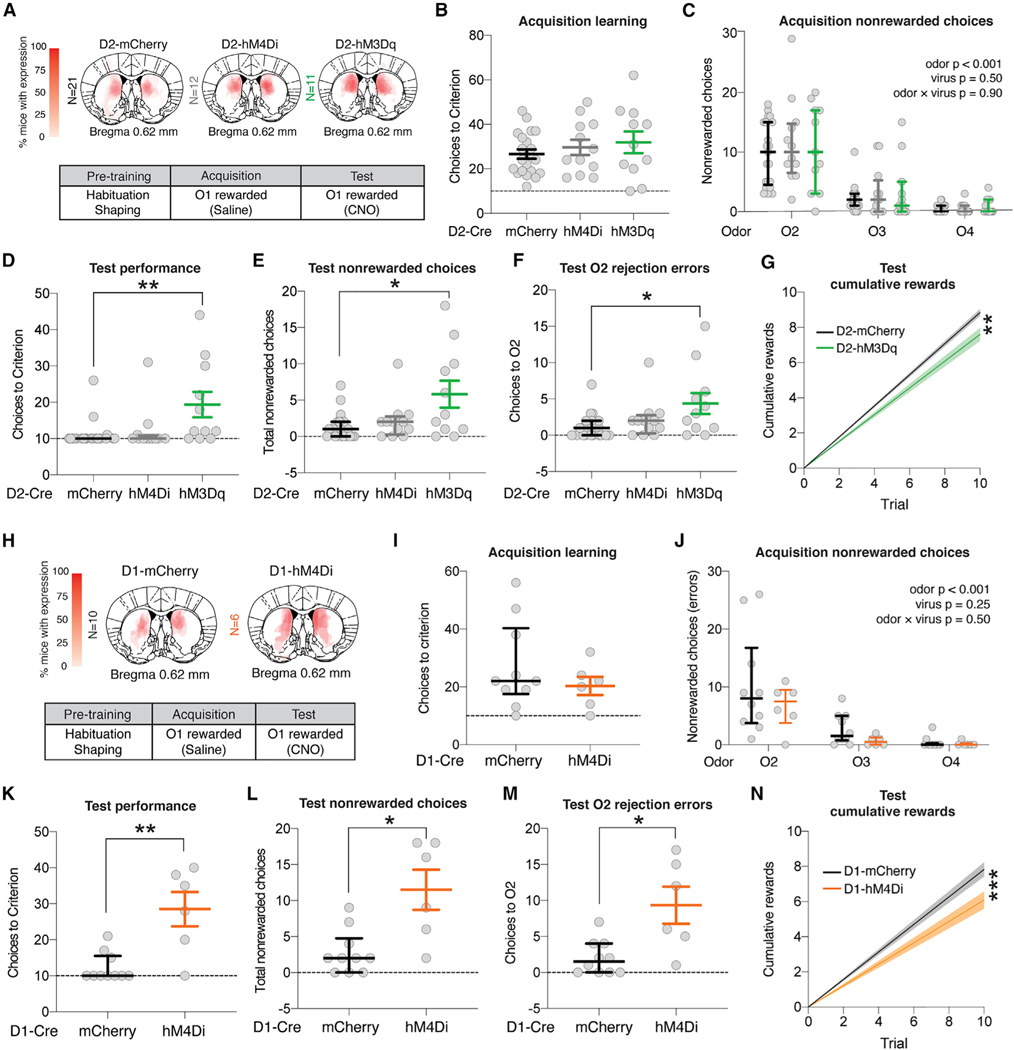
Chemogenetic activation of iSPNs and inhibition of dSPNs both impaired test-phase performance, while inhibition of iSPNs had no significant effects (A) Top panel: injection site and viral spread for D2-Cre DIO-mCherry (N = 21), DIO-hM4Di (N = 12), and DIO-hM3Dq (N = 11) mice. Bottom panel: summary of behavior. (B) Acquisition (saline) choices to criterion. (C) Effect of odor identity (**p < 0.001) and virus (p = 0.50) on nonrewarded choices. (D) Test (CNO) choices to criterion (**p < 0.01). (E) Test nonrewarded choices (*p < 0.05). (F) Test choices to the training-naive preferred odor (O2) (**p < 0.01). (G) Test reward accumulation (**p < 0.01). (H) Top panel: Injection site and viral spread for D1-Cre DIO-mCherry (N = 10) and DIO-hM4Di (N = 6) mice. Bottom panel: summary of behavior. (I) Acquisition (saline) choices to criterion (p = 0.99 Mann-Whitney U test). (J) Effect of odor identity (***p < 0.001) and virus (p = 0.25) on nonrewarded choices. (K) Test choices to criterion (**p < 0.01). (L) Test nonrewarded choices (*p < 0.05). (M) Test choices to the training-naive preferred odor (O2) (*p < 0.05). (N) Test reward accumulation (***p < 0.0001). Mean ± SEM are plotted for normally distributed data. Otherwise, data plotted indicate median ± interquartile range (IQR). Data in (G) and (N) indicate linear regression line with bands plotting the 95% confidence interval. See full statistics in Table S2.

The DMS of D2-Cre mice was bilaterally infused with 0.5 μL of Cre-dependent DREADD virus (hM4Di-mCherry or hM3Dq-mCherry), and mice were trained 4–6 weeks later in the odor-guided serial choice task (Figure 3A). Mice expressing Cre-inducible mCherry were included to control for effects of surgery, adeno-associated virus (AAV) infection, and CNO administration. Prior to acquisition, all mice received intraperitoneal (i.p.) injections of saline (Figure 3A). No difference in acquisition learning—measured as the number of choices to criterion—was observed across groups (ANOVA main effect p = 0.54) (Figure 3B). During acquisition, there was a significant effect of odor identity on nonrewarded choices, with O2 (thyme) being the most frequently chosen nonrewarded odor (ANOVA main effect p < 0.001; Figure 3C). Twenty-four hours after acquisition, all groups were administered CNO (1.0 mg/kg, i.p.) and run in the test phase. mCherry control and D2-Cre inhibitory DREADD (hM4Di) groups exhibited robust recall of the rewarded odor choice and successful rejection of the nonrewarded choices during test, with most mice reaching criterion in the minimum number of trials (Figure 3D). Mice expressing activating DREADD in iSPNs (D2-hM3Dq) took more choices to reach criterion (ANOVA main effect **p < 0.01; Figure 3D), made more nonrewarded choices (ANOVA main effect *p < 0.05; Figure 3E), and made more choices to O2 compared to mCherry controls (ANOVA main effect *p < 0.05; Figure 3F). Given that O2 was the preferred odor prior to acquisition learning, these results suggest that activating iSPNs impaired learned choice rejection during the test phase. Finally, D2-hM3Dq mice were slower to accumulate rewards during the test phase compared to mCherry controls (**p < 0.01; Figure 3G), whereas D2-hM4Di mice did not significantly differ from mCherry controls (Figure S1). These data are inconsistent with predictions of the simple select/suppress heuristic model, in which gains in indirect pathway activity are critical to reject competing low-value options.

### Chemogenetic inhibition of the direct pathway impairs ability to reject nonrewarded choices

We next tested the effect of chemogenetically inhibiting dSPNs on test-phase performance (Figure 3H). Acquisition learning was consistent between groups receiving saline (Figure 3I) (-ANOVA main effect of virus p = 0.25), and again, mice learned to reject the initially preferred O2 (Figure 3J). During the test phase, CNO-treated D1-hM4Di mice showed a significant increase in choices to criterion (**p < 0.01 Mann-Whitney U test; Figure 3K) and total nonrewarded choices (*p < 0.05 Mann-Whitney U test; Figure 3L). D1-hM4Di mice made more choices to O2 compared to D1-mCherry mice (*p < 0.05 Mann-Whitney U test; Figure 3M) and were slower to accumulate rewards (**p < 0.0001; Figure 3N). These results are consistent with the simple select/suppress heuristic, which predicts that disruption of direct pathway activity should reduce selection of O1 during test.

### Trial-by-trial RL modeling suggests that activating iSPNs alters test-phase performance by increasing choice stochasticity

**Figure 4.**
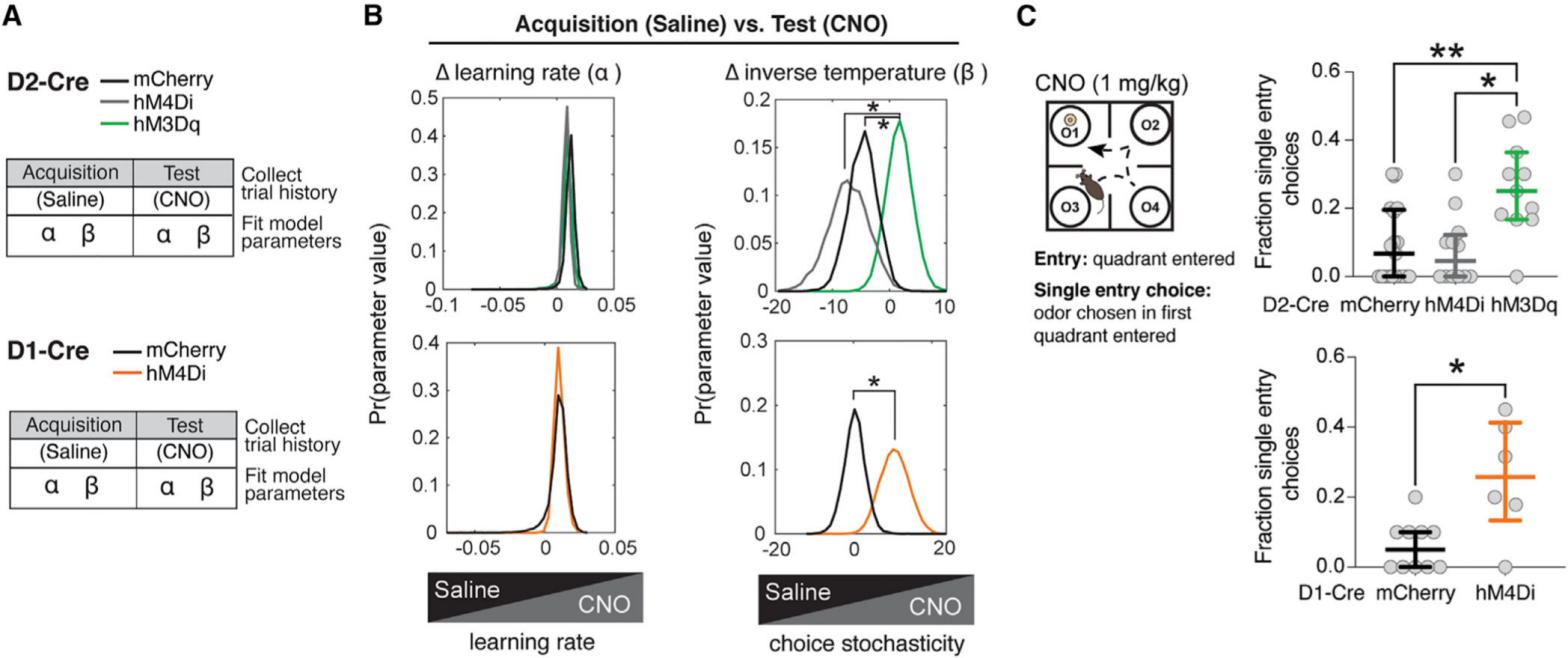
Enhancing iSPN activity and reducing dSPN activity increased choice stochasticity but reduced physical serial exploration of choices before decision (A) RL modeling of acquisition and test performance in D2-Cre (top) and D1-Cre (bottom) groups. (B) Left panel: no effect of chemogenetic manipulation on Δα in D2-Cre (top) or D1-Cre (bottom) groups (p > 0.05). Right panel: chemogenetic activation of iSPNs (green) significantly increased Δβ compared to mCherry control and chemogenetic inhibition of iSPNs (gray). Chemogenetic inhibition of iSPNs did not significantly change Δβ compared to mCherry (top). Chemogenetic inhibition of dSPNs (orange) increased Δβ compared to mCherry control (bottom). (C) Chemogenetic activation of iSPNs (top) (N = 21, 12, 11) or chemogenetic inhibition of dSPNs (bottom) (N = 10, 6) is associated with increased proportion of single entry choices. *p < 0.05, **p < 0.01. Hypothesis tests were conducted using Kruskal-Wallis ANOVA or Mann Whitney U test for behavior and sample-based credible interval for model parameters. See full statistics in Table S2.

To further examine how chemogenetic manipulation affected underlying learning and/or choice processes, we turned to reinforcement learning (RL) models (Daw, 2009). We compared multiple models fit to trial-by-trial changes in behavior using a hierarchical fitting process (Figures 4E and 4H; STAR Methods). Odor values (Q values) were adjusted by a reward prediction error, separate from a selection process that transformed odor values into choice probabilities (see STAR Methods for more details). The best fitting model included phase-specific parameters for learning rate α and inverse temperature parameter β, which captures choice stochasticity (see Table S1 for alternate model comparison and Figure S2 for model validation). Comparing the change in α and β parameters between acquisition and test phases (Δα and Δβ, respectively), we observed that Δα did not differ among D2-Cre groups (Figure 4F), whereas Δβ was significantly higher in mice expressing activating DREADD in iSPNs (D2-hM3Dq) compared to mCherry control (*p < 0.05; 95% credible interval for the group difference [.052, 12.972]) and inhibitory DREADD (D2-hM4Di; *p < 0.05; 95% credible interval for the group difference [.79, 16.97]) (Figure 4G). These data indicate that chemogenetic activation of DMS iSPNs decreases test-phase β, suggesting that enhancing iSPN activity makes choice policy more stochastic or exploratory. RL fits to D1-hM4Di mice similarly showed that Δα was unaffected but that Δβ was significantly higher compared to mCherry control mice (ANOVA main effect *p < 0.05). Importantly, pan-neuronal chemogenetic inhibition (non-Cre-dependent hM4Di) in DMS did not significantly alter test-phase choice strategy according to raw behavioral data or RL-model fits (Figure S3).

We next looked at “entries,” which captures physical exploration of the four quadrants prior to choice (Figure 4A). Mice that expressed activating DREADDs in iSPNs (D2-hM3Dq) or inhibitory DREADDs in dSPNs (D1-hM4Di) had a greater proportion of “single entry choices” in which they dug in the first encountered pot (ANOVA main effect **p < 0.01; Mann Whitney U test *p < 0.05) (Figure 4C). This effect was also seen after pan-neuronal DMS chemogenetic inhibition (Figure S3). There was a significant effect of odor identity on single entry choices in both D2-hM3Dq and D1-hM4Di mice, with rewarded O1 and training-naive preferred O2 most frequently chosen, suggesting that these choices were not random (ANOVA main effect ***p < 0.0001) (Figure S3).

We next examined how chemogenetic manipulation affected task motivation and motor behavior by comparing completed and omitted trials, trial latency, and behavior in the open field and rotarod. All D2-hM3Dq, D2-hM4Di, and D1-hM4Di mice reached test-phase criterion on CNO (Figures 3D and 3K), suggesting that they were capable of performing the task and remained highly motivated to do so. By contrast, mice expressing activating DREADD in dSPNs within DMS (D1-hM3Dq) exhibited a hyperlocomotive phenotype and did not engage in the task during the test phase on CNO (see Video S1). Correct and incorrect choice latency during the test phase did not differ across groups (Figure S4). However, D2-hM3Dq mice omitted significantly more trials during the test phase than D2-hM4Di mice and mCherry controls (ANOVA main effect **p < 0.01; Figure S4). No change in omissions was found for D2-hM4Di or D1-hM4Di mice compared to mCherry controls during the test phase (Mann Whitney U test p = 0.63; Figure S4).

In the open field, CNO administration reduced spontaneous locomotion and decreased vertical rearing in both in D2-hM3Dq and D1-hM4Di groups compared to mCherry controls, though this effect was larger in D2-hM3Dq mice than in D1-hM4Di mice (Figure S4). In the rotarod, performance of D2-hM3Dq mice on CNO was comparable to controls (Figure S4), suggesting that chemogenetic activation of iSPNs did not grossly perturb motor coordination.

## DISCUSSION

Here, we found that chemogenetic activation of iSPNs and chemogenetic inhibition of dSPNs in the DMS disrupted choice rejection in a serial decision-making task. Intriguingly, chemogenetic inhibition of iSPNs had little effect on learned choice rejection, despite evidence of efficacy in other assays. The results from the dSPNs were well predicted by a simple and intuitive select/suppress heuristic of striatal function, but the results from iSPNs contradict the simple assumption that increased activity in this pathway should facilitate learned choice rejection whereas inhibition should disrupt it (Figure S5). Previous studies that examined the striatum’s role in rejecting or avoiding low-value actions (Bryden et al., 2012; Ogasawara et al., 2018; Schmidt et al., 2013) and stimuli (Kim et al., 2017) clearly implicate iSPNs in choice rejection and generally support the assumption that increases in iSPN activity should promote choice rejection. However, our iSPN results are anticipated by previous studies that highlighted the role of iSPNs in action switching in lateralized (Tai et al., 2012; Lee and Sabatini 2021) and nonlateralized contexts (Geddes et al., 2018; Tecuapetla et al., 2016; Nonomura et al., 2018). Our data also complement reported patterns of synaptic plasticity following appetitive goal-directed action learning (Shan et al., 2014), which reveal potentiation onto dSPNs and depression onto iSPNs. Chemogenetic manipulations that impaired choice behavior in our task (dSPN inhibition and iSPN activation) would be predicted to counteract these synaptic changes, potentially interfering with the “read out” of learning-driven plasticity. Several aspects of our task may have enabled us to make surprising observations regarding the role of iSPNs in choice rejection: (1) choices were made serially instead of in parallel as in the case of 2AFC tasks, (2) choice rejection could be “forwardly active” in the sense that it did not require freezing, stopping, or lateral reorientation, and (3) it was acquired much more rapidly than standard operant tasksand thus may rely more heavily on the DMS compared to tasks that require extensive training.

### A role for the indirect pathway in value-based choice exploration

We found that when iSPNs were activated or dSPNs were inhibited, choice became more stochastic/exploratory, meaning that mice were more likely to “explore” (i.e., choose) a lower-value odor as opposed to “exploiting” the highest-value odor, as estimated by RL-model fits (Figure S5). Multiple studies have proposed a role for D2 receptors and the indirect pathway in exploratory choice (Chakravarthy et al., 2010; Lee et al., 2015; Tecuapetla et al., 2016; Lee and Sabatini, 2021). Computational models have also predicted a leading role for D1 receptors and the direct pathway in regulating exploratory choice (Humphries 2012). Using RL models, we found that both iSPN activation and dSPN inhibition had significant effects on the inverse temperature parameter β, consistent with less exploitative, more exploratory choice strategy. These data contradict findings from a recent RL-modeling study of DREADD manipulation of the dorsal striatum SPNs in a two-armed bandit task (Kwak and Jung, 2019). However, our findings are consistent with a working model in which decreases in dopamine or dopamine D2 receptor antagonists (Lee et al., 2015) decrease dSPN activity and/or enhance iSPN activity to promote exploration of alternate choices (Dunovan and Verstynen, 2016; Humphries 2012; Lee and Sabatini, 2021). Notably, this working model of exploration posits that increases in iSPN activity drive choice exploration, in line with our D2-Cre hM3Dq effects.

Our analyses of the inverse temperature parameter β and physical exploration of the arena through entries suggest that stochastic choice exploration and physical exploration are not similarly affected by chemogenetic manipulation of DMS SPNs. Whereas iSPN activation and dSPN inhibition increased stochastic/exploratory choice, physical exploration indexed by arena quadrant entries decreased and was dominated by choices to the first option approached. This observation draws a clear distinction between these two forms of exploration. In this same vein, we may question whether exploration of an alternate choice in 2AFC (Lee et al., 2015; Lee and Sabatini, 2021) or abandoning an ongoing behavior like level pressing (Tecuapetla et al., 2016) and choice in serial decision-making while foraging share common features that can be explained by a single working model.

In future research, comparisons of neural activity across tasks (Bolkan et al., 2022) may clarify how different forms of choice and locomotion (including active rejection, freezing, and stopping) are instantiated. Future models will also benefit from greater levels of detail about cell type, intracellular signaling, local and long-range circuit anatomy, and the opponency between hemispheres.

## Conclusion

Here, we used a serial choice foraging task in which mice could move freely between choices without any requirement for freezing or stopping. Our data largely support existing models of basal ganglia function in which trial-and-error choice drives learning that is later stored or read out in the activity emerging from DMS dSPNs and iSPNs (Bariselli et al., 2019; Peters et al., 2021). Our data confirm in this ethological context that dSPN activity is critical for exploitation of the highest-value option. Contrary to a simple heuristic model of basal ganglia function, increasing, rather than decreasing, iSPN activity disrupts rejection of nonrewarded choices. In our study, iSPN activity promoted more stochastic choice and less, not more, physical exploration of space. We are hopeful that our findings will (1) help define active choice rejection as a critical behavior separate from freezing and stopping and (2) aid in the design of new therapies for addiction and other conditions in which greater capacity for active choice rejection is desirable.

### Limitations of the study

Our study used chemogenetics to alter DMS neural activity. Manipulation was restricted to DMS Cre-expressing neurons within D1- and D2-Cre BAC transgenic mice but was still coarse in that it did not target specific ensembles of neurons activated by specific odor cues. This limits our interpretation of how learned choice rejection is achieved by specific dSPN and iSPN neural ensembles. In addition, while we observed low rates of D2R-Cre-mediated DREADD expression in cholinergic interneurons, we did not rule out that DREADD was expressed in other interneuron populations. Next, chemogenetic inhibition of DMS iSPNs did not impair choice rejection, a negative result. Our control experiments indicate that chemogenetic inhibition of iSPNs altered behavior and neurotransmission (Figure 2), and we observed significant effects of chemogenetic inhibition of dSPNs on choice (Figure 3). Despite this evidence of efficacy, we cannot rule out the possibility that more robust methods of inhibition of DMS iSPNs would disrupt choice rejection.

We observed significant effects of DMS chemogenetic manipulation on spontaneous locomotion in open field (Figure S4), which may have contributed to choice behavior in a nonspecific way. However, locomotor and choice effects of striatal manipulations may be impossible to dissociate if SPNs multiplex aspects of choice, accuracy, cost, and/or effort (Baraduc et al., 2013; Mazzoni et al., 2007; Hamid et al., 2016; Wang et al., 2013; Mourra et al., 2020). Future experiments that manipulate alternative choice value and discriminability should further inform these observations.

## STAR★METHODS

### RESOURCE AVAILABILITY

#### Lead contact

Further information and requests for resources and reagents should be directed to and will be fulfilled by the lead contact, Dr. Linda Wilbrecht (wilbrecht@berkeley.edu).

#### Materials availability

This study did not generate new unique reagents.

#### Data and code availability

- Serial choice task data is available on GitHub: https://github.com/kdelevich/CELL-REPORTS-D-20-04005. Other data reported in this paper will be shared upon request to the lead contact.
- Hierarchical RL model code is available on GitHub: https://github.com/kdelevich/CELL-REPORTS-D-20-04005. DOIs are listed in the key resources table.
- Any additional information required to reanalyze the data reported in this paper is available from the lead contact upon request.

### EXPERIMENTAL MODEL AND SUBJECT DETAILS

All mice were weaned on postnatal day (P)21 and group-housed on a 12:12hr reverse light:dark cycle (lights on at 10PM). C57BL/6 BAC transgenic mice expressing Cre recombinase under the regulatory elements for the D1 and D2 receptor (Drd1a-Cre and D2-Cre ER43) were obtained from Mutant Mouse Regional Resource and bred in our colony. Mice had *ad libitum* access to food and water before food restriction in preparation for training. All procedures were approved by the Animal Care and Use Committee of the University of California, Berkeley and complied with the NIH guide for the use and care for laboratory animals. For all behavior experiments, adult male and female mice were injected at 6–8 weeks of age and behavioral assays or electrophysiological recordings were performed at 10–16 weeks of age.

### METHODS DETAILS

#### Viruses and tracers

Adeno-associated viruses (AAVs) were produced by the Gene Therapy Center Vector Core at the University of North Carolina at Chapel Hill or by Addgene viral service and had titers of >10^12^ genome copies per mL. For chemogenetic manipulations, mice were bilaterally injected with 0.5 uL of rAAV8-hsyn-DIO-mCherry, rAAV8-hsyn-DIO-hM3Dq-mCherry, or rAAV8-hsyn-DIO-hM4Di-mCherry. For rotational bias experiments, mice were unilaterally injected with 0.5 uL of rAAV8-hsyn-DIO-mCherry and 0.5 uL of rAAV8-hsyn-DIO-hM3Dq-mCherry or rAAV8-hsyn-DIO-hM4Di-mCherry in the opposite hemisphere. For *in vitro* electrophysiological validation experiments of rAAV8-hsyn-DIO-hM4Di-mCherry, mice were bilaterally injected with 0.69 uL of a 2:1 mixture of rAAV8-hsyn-DIO-hM4Di-mCherry and rAAV5-Ef1α-DIO-hChR2-EYFP. For *in vitro* electrophysiological validation experiments of rAAV8-hsyn-DIO-hM3Dq-mCherry, mice were bilaterally injected with 0.5 μL of rAAV8-hsyn-DIO-hM3Dq.

#### Stereotaxic virus injection

Male and female mice (6–8 weeks) were deeply anesthetized with 5% isoflurane (vol/vol) in oxygen and placed into a stereotactic frame (Kopf Instruments; Tujunga, CA) upon a heating pad. Anesthesia was maintained at 1–2% isoflurane during surgery. An incision was made along the midline of the scalp and small burr holes were drilled over each injection site. Virus or tracer was delivered via microinjection using a Nanoject II injector (Drummond Scientific Company; Broomall, PA). Injection coordinates for DMS were (in mm from bregma): 0.90 anterior, +/− 1.4 lateral, and −3.0 from surface of the brain. Mice were given subcutaneous injections of meloxicam (10 mg/kg) during surgery and 24 & 48 h after surgery. Mice were group-housed before and after surgery and 4–6 weeks were allowed for viral expression before behavioral training or electrophysiology experiments.

#### Drugs

Clozapine-N-Oxide was generously provided by the NIMH Chemical Synthesis and Drug Supply Program (NIMH C-929). CNO was made fresh each day and dissolved in DMSO (0.5% final concentration) and diluted to 0.1 mg/mL in 0.9% saline USP. Tetrodotoxin (TTX), DL-AP5, and NBQX disodium salt were purchased from Tocris Biosciences (Ellisville, MO).

#### Electrophysiology

Mice were deeply anesthetized with an overdose of ketamine/xylazine solution and perfused transcardially with ice-cold cutting solution containing (in mM): 110 choline-Cl, 2.5 KCl, 7 MgCl2, 0.5 CaCl2, 25 NaHCO3, 11.6 Na-ascorbate, 3 Na-pyruvate, 1.25 NaH2PO4, and 25 D-glucose, and bubbled in 95% O2/5%CO2. 300 μm thick sections (sagittal for optogenetic stimulation experiment, coronal for all others) were cut in ice-cold cutting solution before being transferred to ACSF containing (in mM): 120 NaCl, 2.5 KCl, 1.3 MgCl2, 2.5 CaCl2, 26.2 NaHCO3, 1 NaH2PO4 and 11 Glucose. Slices were bubbled with 95% O2/5% CO2 in a 37°C bath for 30 min, and allowed to recover for 30 min at room temperature before recording. All recordings were made using a Multiclamp 700B amplifier and were not corrected for liquid junction potential. The bath was heated to 32°C for all recordings. Data were digitized at 20 kHz and filtered at 1 or 3 kHz using a Digidata 1440 A system with pClamp 10.2 software (Molecular Devices, Sunnyvale, CA, USA). Only cells with access resistance of <25 MΩ were retained for analysis. Access resistance was not corrected. Cells were discarded if parameters changed more than 20%. Data were analyzed using pClamp or R (RStudio 0.99.879; R Foundation for Statistical Computing, Vienna, AT).

Spontaneous spiking in GPe neurons was recorded in cell-attached configuration. To evoke synaptic transmission by activating ChR2, we used a single wavelength LED system (470 nm; Thorlabs; Newtown, NJ) connected to the epifluorescence port of the Olympus BX51 microscope. Light pulses of 1–10 ms triggered by a TTL (transistor-transistor logic) signal from the Clampex software (Molecular Devices; Sunnyvale, CA) were delivered through a 63× objective and used to evoke synaptic transmission. Blue light pulses were delivered once every 10 s, and a minimum of 30 trials were collected. Light-evoked IPSCs were recorded in whole-cell configuration at +10 mV holding potential in the presence of DL-AP5 (50 μM) and NBQX disodium salt (33 μM) to block glutamatergic neurotransmission. Recording pipettes had 2.5–5.5 MΩ resistances and were filled with internal solution (in mM): 115 Cs-methanesulfonate, 10 HEPES, 10 BAPTA, 10 Na2-phosphocreatine, 5 NaCl, 2 MgCl2, 4 Na-ATP, 0.3 Na-GTP.

Whole-cell current clamp recordings were performed using a potassium gluconate-based intracellular solution (in mM): 140 K Gluconate, 5 KCl, 10 HEPES, 0.2 EGTA, 2 MgCl2, 4 MgATP, 0.3 Na2GTP, and 10 Na2-Phosphocreatine. For current clamp recordings to validate CNO induced depolarization in Gq-DREADD- expressing Drd2+ neurons, ACSF contained 0.5 μM TTX and a stable baseline was collected for 3–5 min before ACSF containing 0.5 μM TTX +10 μM CNO was washed on. For all electrophysiology experiments, both male and female mice were used.

#### Behavioral assays

Adult male and female mice (10–16 weeks) were used in behavioral assays. Mice were first tested in 4 choice odor-guided serial choice task and then ≥2 weeks later were tested in locomotor and/or rotarod tasks so that performance on CNO could be compared within animals across tasks. Prior to all behavior assays, mice were habituated to the testing room for 30 min, and all behavior testing began 30 min after CNO treatment. Importantly, all groups (including DIO-mCherry) were administered CNO to control for potential off-target effect of the CNO metabolite clozapine (Mahler and Aston-Jones, 2018).

#### Rotational bias assay

Mice were placed in a cylinder (Nalgene) in a dark sound-attenuated box for a 15 min habituation session. The next day, mice were injected with 1 mg/kg CNO or saline and placed in the cylinder for 15 min, during which they were recorded on an infrared camera. The following day, mice received CNO or saline and were tested again. Mice were tested for two rounds of counterbalanced CNO and saline administration separated by one week. Cylinders were cleaned between animals with water and acetic acid. Videos were analyzed by an experimenter blind to condition. Briefly, videos were analyzed using the open source deep-learning based framework for estimating positions of animal body parts, SLEAP (Pereira et al., 2022). To provide initial training data for the algorithm, 30 frames were randomly selected from 15 min (approximately 22000 frames, 15 frames/s) of video data. Head, torso, and tail base were manually labeled. After inferences generated by the model reached a satisfactory accuracy and its performance plateaued, the model was used to generate head, torso, and trailhead coordinates for the remaining frames in the dataset. Any missing values were substituted with the nearest non-empty values. Node (head, torso, and tail-head) coordinates were filtered using a Savitzky-golay filter (Polynomial Degree = 3, Window Length = 15) to achieve smooth tracking. The first 5 frames were dropped to exclude unstable video captures. To quantify rotation, head coordinates were first translated to egocentric coordinates by subtracting the torso coordinates from it. Rotation angle was calculated as the change in angle between the egocentric head-torso vector and the positive x axis, with the previous torso node being the origin. Calculation artifacts from when the animal crossed the positive x axis were replaced by the nearest valid angle. A gaussian filter (sigma = 3) was applied to smooth the calculated angles. Tail-head displacement and rotation were used to identify moving versus non-moving states, and tail-head displacement for each frame was calculated as the Euclidean distance between the coordinates of the current frame and the coordinates of the previous frame. A frame is classified as moving if the corresponding tail-head displacement was greater than 0.4 pixels and if the rotation angle is greater than 0.5, or if rotation angle is greater than 2 to account for robust head swings. Only frames that were classified as moving were used to calculate rotation bias.

##### 4 choice odor-guided serial choice task

The odor-guided serial choice task used has previously been described in detail (Johnson et al., 2016). In this task only the odor cue is predictive, and spatial or egocentric information are irrelevant. This behavior is also ethologically relevant because mice use odor information to locate food sources (Howard et al., 1968). Briefly, mice were food restricted to ~85% bodyweight prior to training. On day 1, mice were habituated to the testing arena, on day 2 were taught to dig for cheerio reward in a pot filled with unscented wood shavings, on day 3 underwent a 4-choice odor discrimination in which they acquire the rule that 1 of 4 presented odors is rewarded (acquisition), and finally on day 4 were tested for recall of the previously learned odor-reward association (test) (Figure 1A). During the acquisition phase of the task, mice learned to discriminate among four pots with different scented wood shavings (anise, clove, litsea and thyme). All 4 pots were sham-baited with cheerio (under wire mesh at bottom) but only one pot was rewarded (anise). The pots of scented shavings were placed in each corner of an acrylic arena (12″, 12″, 9″) divided into 4 quadrants. Mice were placed in a cylinder in the center of the arena, and a trial started when the cylinder was lifted. Mice were then free to explore the arena until a choice was signaled by a bimanual dig to the wood shavings. The cylinder was lowered as soon as a choice was made. If the choice was incorrect, the trial was terminated and the mouse was gently encouraged back into the start cylinder. Trials in which no choice was made within 3 min were considered omissions. If mice omitted for 2 consecutive trials, they received a reminder: a baited pot of unscented wood shavings was placed in the center cylinder and mice dug for the “free” reward. Mice were disqualified if they committed 4 pairs of omissions. The location of the 4 odors was shuffled on each trial, and criterion was met when the mouse completed 8 out of 10 consecutive trials correctly. 24-h after completing the acquisition phase, mice underwent a recall test of the initial odor-reward rule to criterion. For chemogenetic manipulation experiments, mice were injected with saline 30 min prior to acquisition training and injected with CNO (1.0 mg/kg) 30 min prior to the test phase. During acquisition and test phase, experimenters (blind to group) manually scored entries into each quadrant, latency to dig, and odor choices. mCherry mice (D2-mCherry or D1-mCherry) were run in parallel with DREADD mice of the same genotype.

##### Rotarod test

On day 1, mice underwent a habituation trial in which they were placed individually in a clean holding cage for 5 min. The rotarod (47650 Rota-Rod NG Ugo Basile; Monvalle VA, Italy) was then set at 5 rpm constant speed and each mouse was placed on the rod for 1 min. The mice were then returned to the holding cage for another 5 mins before initiating the first trial. Each session consisted of 5 trials in which the rotarod constantly accelerated from 5–40 rpm over a period of 300 s, and the latency at which mice fell off the or held onto the rod for a full rotation was recorded. Mice rested for 5 mins in the holding cage between trials. Asymptotic performance was reached by day 3 of training (Figure S4). On day 4, DIO-DREADD and DIO-mCherry mice were administered CNO (1 mg/kg, i.p.) 30 min before rotarod testing began. On day 5 mice were tested drug-free in rotarod performance. Females and males were run during separate sessions. The rotarod apparatus was cleaned between mouse cohorts with 3% hydrogen peroxide (for plastic components) and 70% ethanol (for metal troughs).

##### Open field locomotor assay

On day 1, mice underwent a habituation session in which they were placed in a clear acrylic box (225 × 225 mm) inside a sound attenuated chamber (Med Associates; Fairfax, VT) with lights off. Locomotion was monitored for 15 min using infrared beam breaks (Versamax, AccuScan Instruments, Columbus, OH). On days 2 and 3 mice received injections of saline or CNO (counterbalanced across mice) 30 min before their locomotion was monitored for 15 min. The chamber was cleaned with 70% ethanol between mice.

#### Histology

Mice were transcardially perfused with PBS followed by 4% PFA in PBS. Following 24 h postfixation, coronal brain slices (75 μm) were sectioned using a vibratome (VT100S Leica Biosystems; Buffalo Grove, IL). To confirm viral targeting, we performed a standard immunohistochemical procedure using a primary antibody against red fluorescence protein (RFP) (rabbit, Rockland 600–401-379; 1:1000) to enhance the mCherry signal expressed in mice transduced with rAAV8-hSyn-DIO-DREADD-mCherry or rAAV8-hSyn-DIO-mCherry. For ChAT colocalization experiments, a primary antibody against ChAT (1:1000 Millipore) was incubated overnight. The next day, sections were stained for 2 h in corresponding secondary antibodies. Sections were counterstained with DAPI (Life Technologies; Carlsbad, CA). Images were acquired with a Zeiss Axio Scan.Z1 epifluorescence microscope (Molecular Imaging Center, UC Berkeley) at 10× magnification and viewed using FIJI (ImageJ). For colocalization experiments, mCherry signal was enhanced as previously described, and images were acquired using a Zeiss LSM 710 confocal microscope (Biological Imaging Facility, UC Berkeley). Anatomical regions were identified according to the Mouse Brain in Stereotaxic Coordinates by Franklin and Paxinos and the Allen Institute Mouse Brain Atlas.

#### RL model

We modeled acquisition and test phase behavior using a reinforcement learning model driven by an iterative error-based rule (Rescorla and Wagner, 1972; Sutton and Barto, 1998). The model uses a prediction error (δ) to update the value (Q) of each odor stimulus, where d is the difference between the experienced feedback (λ) and the current expected value (r = 100 for rewarded, r = 0 for unrewarded) scaled by a learning rate parameter (α), with 0<α<1:

$$Q_{t+1}(choice)=Q_{t}(choice)+\alpha \times \delta_{t}$$

$$\delta_{t}=r_{t}-Q_{t}(choice)$$

$$r_{t}=\{\begin{array}{l} 100\;if\;rewarded\;choice\\ else\;0 \end{array}$$

Because mice exhibit innate preferences for odors, we set initial odor values to fixed shared parameters [v1,v2,v3,v4] for all mice tested (see Johnson et al., 2016); these parameters were estimated at the group level within the hierarchical model. To model trial-by-trial choice probabilities, the stimulus values were transformed using a softmax function to compute choice probabilities based on estimated odor values, $Q\left(O_{i}\right)$: The inverse temperature parameter (β), which we refer to in the text as the explore/exploit parameter, determined the stochasticity of the choices:

$$\sigma \left(Q\left(O_{i}\right)\right)=\frac{e^{\beta Q\left(O_{i}\right)}}{\sum_{i=1}^{n}e^{\beta Q\left(O_{i}\right)}}$$

We used hierarchical Bayesian model fitting to infer the best fitting parameters, using the package STAN in Matlab (Carpenter et al., 2017). We assumed that odor values were shared by all animals, and that other parameters (αand β for each phase) were drawn from group level distributions defined by the experimental manipulation. Hierarchical Bayesian modeling provides a direct measure of uncertainty on group parameter estimates by outputting samples that create a distribution, which enables direct statistical testing by interrogating this distribution. Thus, as is standard practice, we performed statistical tests on the distribution of samples obtained for the group-level hyperparameters – for example, we identified that Δβ was significantly greater than 0 if the 95% credible interval of the distribution of this variable in the samples did not include 0. We compared the alternative models using the WAIC (Vehtari et al., 2017; Watanabe, 2010) and found that the best fit model included phase-specific (non-zero) α and β parameters; all RL model comparisons are presented in Table S1. To assess model performance, trial-by-trial behavioral data was simulated using the best fit parameters for each animal, and average simulated choices to criterion for acquisition and test phases (100 simulations/animal) were plotted against the actual choices to criterion for each animal - see Figure S2.

### QUANTIFICATION AND STATISTICAL ANALYSIS

Statistical tests (excluding model parameters) were performed in GraphPad Prism 7.0 (San Diego, CA) and the R programming environment. Groups were compared using one-way ANOVA if data were normally distributed or Kruskal-Wallis ANOVA if data were not normally distributed. When the ANOVA yielded significant results (p < 0.05), a post-hoc Tamhane’s T2 test or Dunn’s test was used to compare DREADD manipulation groups to the mCherry control group and D2-hM4Di and D2-hM3Dq groups. In several cases, D2-hM3Dq and D1-hM4Di groups were also compared. All hypothesis testing was corrected for multiple comparisons. Data from D2-mCherry and D1-mCherry groups were pooled into a single ‘mCherry’ group for analysis presented in Figure S4. Full statistical test information and animal numbers are presented in Table S2.

## Supplementary Material

1

2

3

## Figures and Tables

**Table:** KEY RESOURCES TABLE

REAGENT or RESOURCE	SOURCE	IDENTIFIER

Antibodies		

rabbit anti-RFP Antibody Pre-adsorbed	Rockland	Cat# 600–401-379; RRID:AB_2209751
goat anti-choline acetyltransferase	Millipore	Cat# AB144P; RRID:AB_2079751

Bacterial and virus strains		

pAAV-hSyn-DIO-mCherry	Addgene	50459-AAV8
pAAV-hSyn-DIO-hM3D(Gq)-mCherry	Addgene	44361-AAV8
pAAV-Ef1a-DIO hChR2(E123T/T159C)-EYFP	Addgene	35509-AAV5
pAAV-hSyn-DIO-hM4D(Gi)-mCherry	Addgene	44362-AAV8

Chemicals, peptides, and recombinant proteins		

Clozapine-N-oxide	NIMH Chemical Synthesis and Drug Supply Program	C-929
Tetrodotoxin	Tocris	1069
DL-AP5	Tocris	0105
NBQX disodium salt	Tocris	1044
Anise extract	McCormick	BHBUST051718A2964
Clove	San Francisco Massage Supply Co.	N/A
Litsea	San Francisco Massage Supply Co.	N/A
Thymol “thyme”	Alfa Aesar	A14563
Honey nut cheerios	General Mills	N/A

Deposited data		

Odor-guided serial choice task data.	https://github.com/kdelevich/CELL-REPORTS-D-20-04005	https://doi.org/10.5281/zenodo.6609046

Experimental models: Organisms/strains		

D2-Cre (ER43)	MMRRC	017268-UCD
D1-Cre (EY217)	MMRC	030779-UCD
C57Bl/6	Charles River	027

Software and algorithms		

SLEAP v1.1.5	https://sleap.ai/	https://github.com/murthylab/sleap
Hierarchical RL model code	https://github.com/kdelevich/CELL-REPORTS-D-20-04005	https://doi.org/10.5281/zenodo.6609046
pClamp 10	Molecular Devices	https://www.moleculardevices.com/
Prism (9.3.1)	Graphpad software	https://www.graphpad.com
Illustrator (26.3.1)	Adobe	https://www.adobe.com
Excel (16.60)	Microsoft	https://www.microsoft.com/en-us/microsoft-365/excel

## Highlights

- Inhibiting iSPNs in the DMS does not impair learned, active choice rejection
- Activating iSPNs or inhibiting dSPNs in the DMS impairs active choice rejection
- Activating iSPNs or inhibiting dSPNs also promotes stochastic, exploratory choice
