# Supplementary material for: Activation, but not inhibition, of the indirect pathway disrupts choice rejection in a freely moving, multiple-choice foraging task
Source: Cell Rep. Author manuscript; Available in PMC 2023 Sep 6. (PMC10481643; doi:10.1016/j.celrep.2022.111129)
Supplement: 1 [file NIHMS1829251-supplement-1.pdf]

Cell Reports, Volume *40*

Supplemental information

Activation, but not inhibition, of the indirect pathway disrupts choice rejection in a freely moving, multiple-choice foraging task

Kristen Delevich, Benjamin Hoshal, Lexi Z. Zhou, Yuting Zhang, Satya Vedula, Wan Chen Lin, Juliana Chase, Anne G.E. Collins, and Linda Wilbrecht

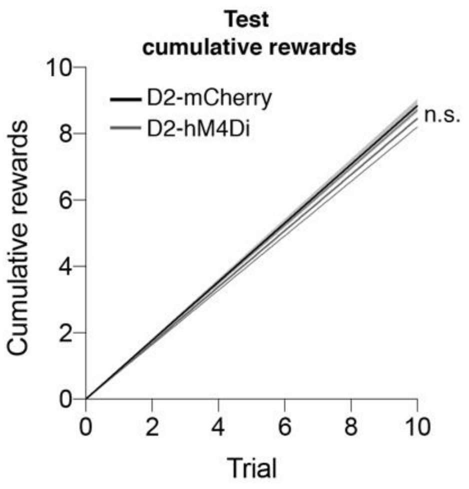

Fig. S1 | iSPN chemogenetic inhibition did not significantly affect test phase reward accumulation compared to D2-mCherry control mice. Related to Figure 3. Linear regression lines fit to first 10 choices of Test Phase with 95% confidence bands plotted. D2-mCherry and D2-hM4Di regression slope estimates overlapped at the 95% confidence interval (N=21, 12).

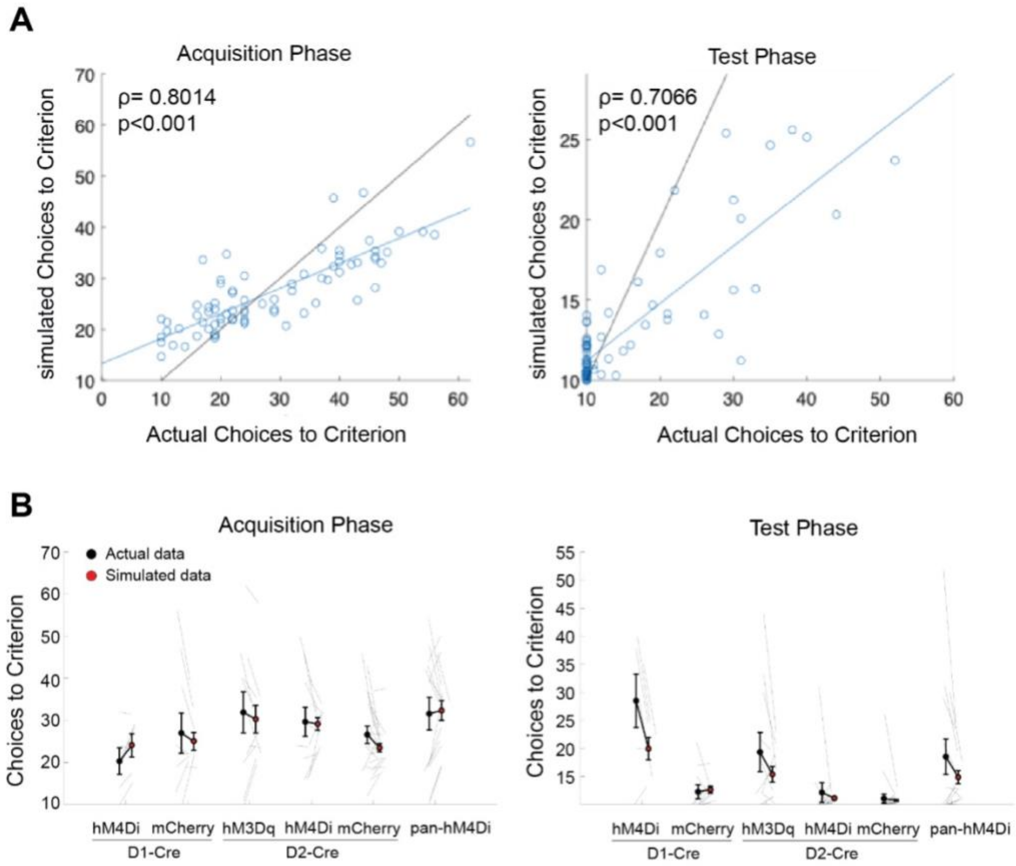

Fig. S2 | RL model validation. Related to Figure 4. (A) Using best fit phase-specific α and β parameters for each animal, trial histories were simulated. The average choices to criterion (100 simulations) for each mouse (N=60) was plotted against its actual choices to criterion for acquisition (left) and test (right) phases. Spearman's correlation coefficient across all animals was significant in acquisition phase (ρ=0.80, p<0.001) and test phase (ρ=0.71, p<0.001). **(B)** Average simulated choices to criterion vs. actual choices to criterion shown for acquisition (left) and test (right) phases by experimental group replicate group differences in test phase and absence of group differences in acquisition phase. Each line represents individual mouse. Data shown as Mean ± SEM.

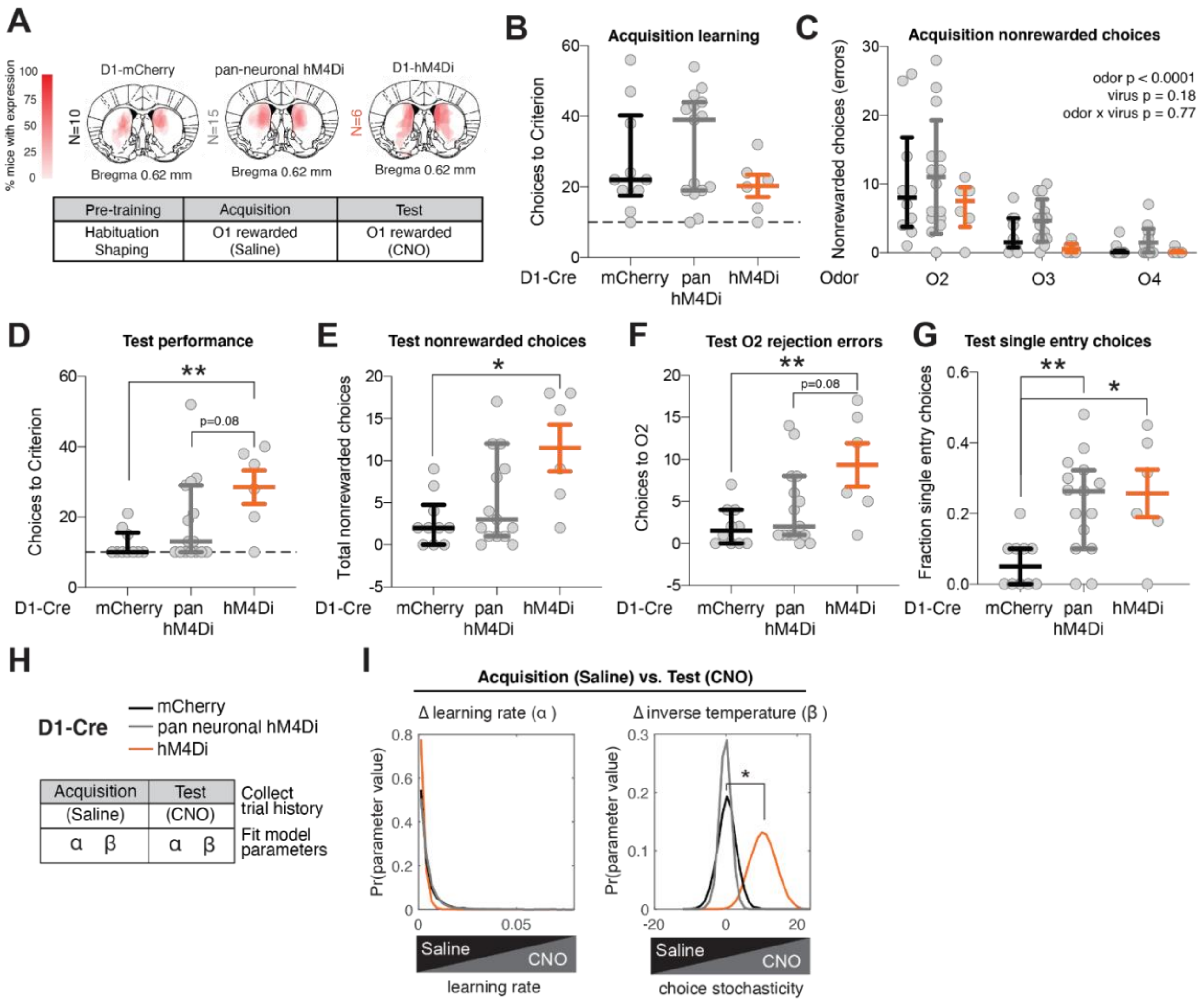

Fig. S3 | D1-Cre restricted but not pan-neuronal chemogenetic inhibition within DMS altered choice strategy. Related to Figures 3 and 4. (**A**) Top panel: schematic illustrating injection site and viral spread D1-Cre DIO-mCherry (N=10), hSyn-hM4Di (n=15), and DIO-hM4Di (N=6) mice. Opacity indicates the number of mice in each cohort that expressed virus in a given location. Bottom panel: summary of behavioral training and drug administration for D1-Cre groups. (**B**) Acquisition (saline) choices to criterion (p=0.50 Kruskal-Wallis ANOVA). **(C)** Effect of odor identity ($F(1.23, 31.52)= 31.70$, ***$p<0.0001$ two-way ANOVA) but not virus ($F(2, 28)= 1.84$, $p=0.18$ two-way ANOVA) on nonrewarded choices was significant. **(D)** Test (CNO) choices to reach criterion (**$p<0.01$ Kruskal-Wallis ANOVA with Dunn's test for multiple comparisons). **(E)** Test nonrewarded choices (*$p<0.05$ Kruskal-Wallis ANOVA with Dunn's test for multiple comparisons). **(F)** Test choices to the training-naïve preferred odor (O2) (**$p<0.01$ Kruskal-Wallis ANOVA with Dunn's test for multiple comparisons). **(G)** Both pan-neuronal and dSPN selective chemogenetic inhibition increased fraction of single entry choices (*$p<0.05$, **$p<0.01$ Kruskal-Wallis ANOVA with Dunn's test for multiple comparisons). **(H)** RL modeling of acquisition and test performance in D1-Cre groups with Cre-dependent (DIO-mCherry; DIO-hM4Di) or pan neuronal (hSyn-hM4Di) viral expression. (**I**) $\Delta\alpha$ and $\Delta\beta$ parameters track change in decision parameters between acquisition and test phases. Left panel: No effect of chemogenetic manipulation on $\Delta\alpha$ in D1-Cre groups ($p>0.05$). Right panel: Chemogenetic inhibition of dSPNs

(orange) significantly increased $\Delta\beta$ compared to mCherry control (bottom). Pan-neuronal chemogenetic inhibition of DMS did not change $\Delta\beta$ compared to mCherry control ($p>0.05$).

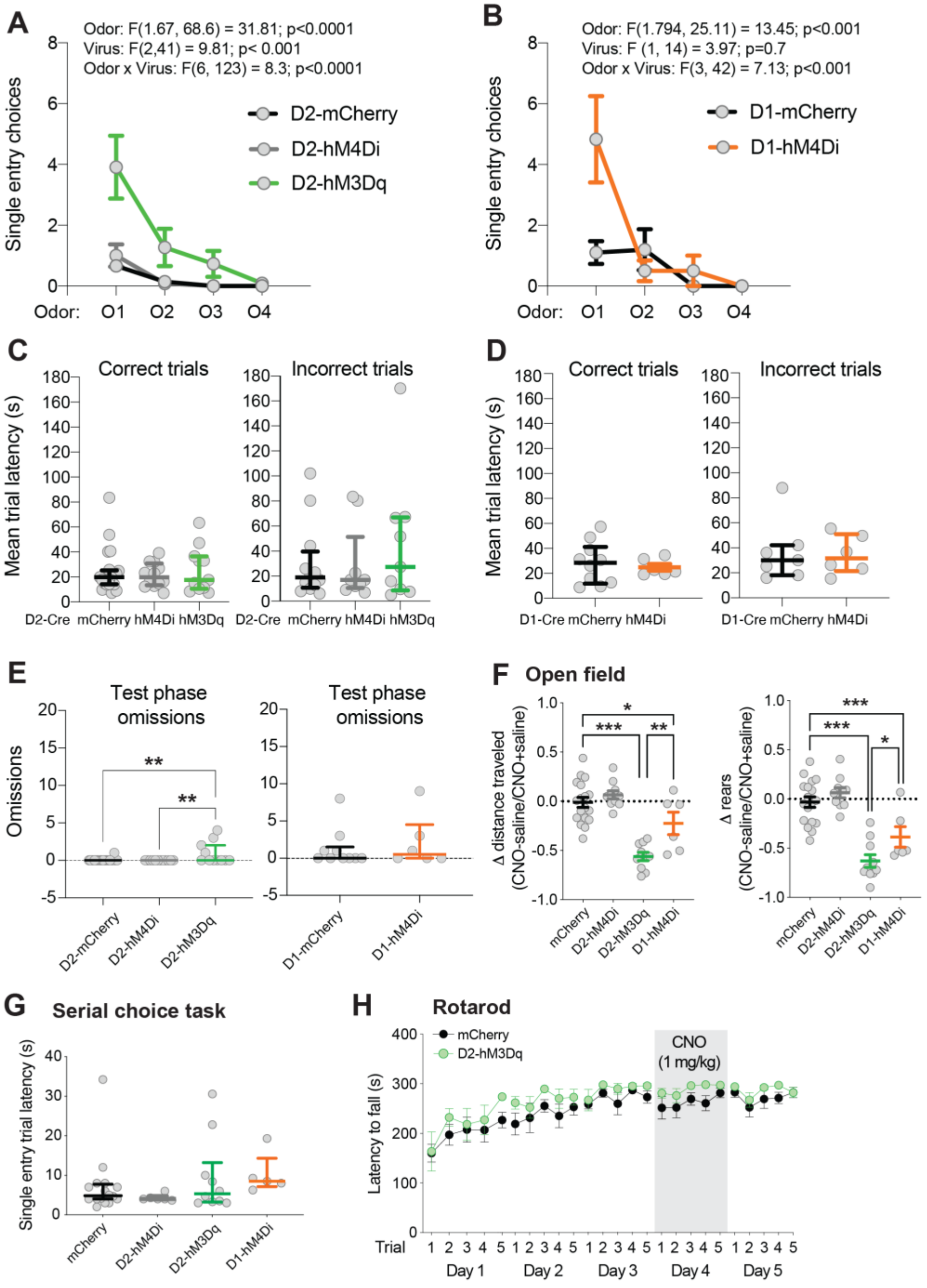

Fig. S4 | Chemogenetic manipulation effects on single entry choices, trial latencies, omissions, and locomotion outside of the task context. Related to Figure 3. (**A**) Chemogenetic activation of iSPNs (D2-hM3Dq) increases the number of single entry choices during Test phase in an odor identity dependent manner (N= 21, 12, 11). (**B**) Chemogenetic inhibition of dSPNs (D1-hM4Di) increases the number of single entry choices during Test phase in an odor identity-dependent manner (N= 10, 6). O1 = rewarded odor 1; O2 = training naïve preferred odor. (**C**) There was no significant effect of virus on Test phase choice latency for correct trials (left; p= 0.98 Kruskal-Wallis ANOVA) or incorrect trials (right; p= 0.90 Kruskal-Wallis ANOVA). (**D**) There was no

significant effect of virus on Test phase choice latency for correct trials (left; $p>0.99$ Mann-Whitney U test) or incorrect trials (right; $p= 0.95$ Mann-Whitney U test. (**E**) Left panel: Effect of virus on Test Phase omissions (no choice made during 3 minute trial) in D2-Cre mice (**$p<0.01$; Kruskal-Wallis ANOVA); right panel: Effect of virus on Test phase omissions in D1-Cre mice ($p= 0.63$; Mann-Whitney U test). (**F**) Left panel: spontaneous locomotion was significantly reduced in D2-hM3Dq and D1-hM4Di mice on CNO (1 mg/kg) compared to saline ($F(3,40)= 22.89$, ***$p<0.0001$, one-way ANOVA). Right panel: CNO administration significantly reduced the number of vertical rears made by D2-hM3Dq and D1-hM4Di mice ($F(3,40)= 24.33$ ***$p<0.0001$, one-way ANOVA). (**G**) There was no significant effect of virus on single entry trial latency ($p=0.11$; Kruskal-Wallis ANOVA). (**H**) Chemogenetic activation of iSPNs (D2-hM3Dq) did not significantly affect latency to fall on the rotarod after stable performance was attained (main effect of drug compared across days 3-5 $F(2, 30) = 0.09$, $p=0.91$, two-way repeated measures ANOVA). Data are presented as Mean ± SEM in panels A-B, F, H and Median ± IQR in panels C-E and G. Post hoc comparisons *$p<0.05$, **$p<0.01$, ***$p<0.001$ uncorrected Fisher's LSD.

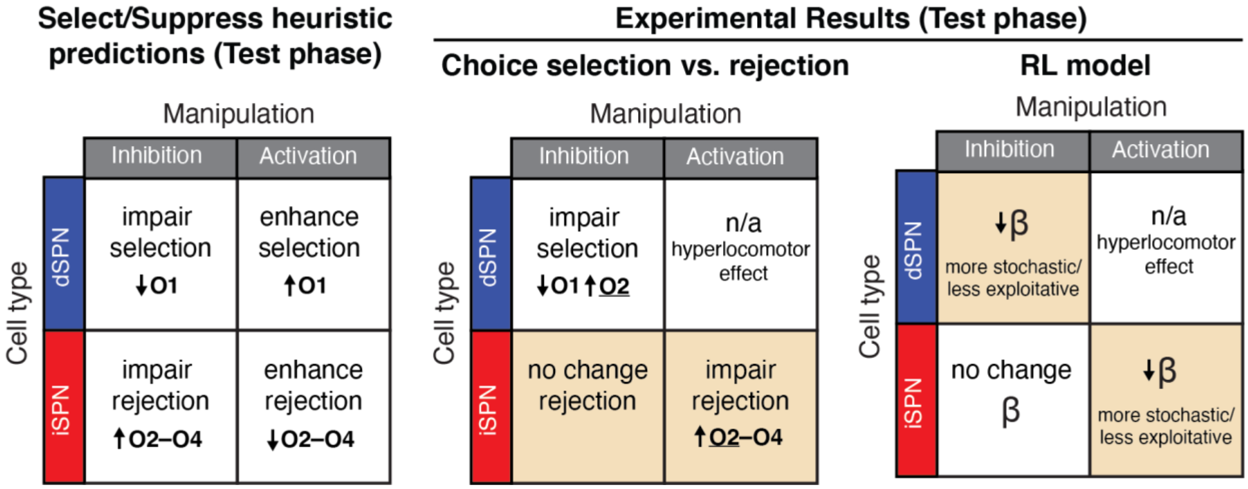

Fig. S5 | Comparison of Select/Suppress heuristic predictions with pathway-specific chemogenetic manipulation results. Related to Figures 1, 3, and 4. The select/suppress heuristic of striatal function emphasizes the role of iSPNs in choice rejection. Therefore, we hypothesized that iSPN inhibition would impair choice rejection, leading to increased choices to nonrewarded odors (O2–O4) and higher choices to criterion. dSPN inhibition was expected to impair choice selection which would similarly lead to more choices to criterion and suboptimal selection of nonrewarded choices (left panel). Our experimental results (center panel) contradict the select/suppress predictions because iSPN inhibition did not affect choice rejection whereas iSPN activation impaired choice rejection, as seen by an increase in nonrewarded choices (O2–O4). Finally, we made unique insights into the role of DMS dSPNs and iSPNs in choice behavior using RL models. We found that either dSPN activation or iSPN inhibition decreased the inverse temperature parameter β (right panel).

model	Learning rate α	Softmax β	Phase decay	Number of parameters per subject	WAIC
ab	single	single	-	2	5682
abf	single	single	x	3	5670
abb	single	per phase	-	3	5663
aabf	per phase	single	x	4	5635
**aabb**	**per phase**	**per phase**	**-**	**4**	**5622**
a0bb	per phase; α_{recall}=0	per phase	-	3	5916

Supplementary Table 1 | RL model comparison. Related to Figure 4.
We tested multiple instantiations of the model to capture task phase effects. Specifically, we investigated whether behavior would be better captured by shared or separate learning rate and softmax inverse temperature across phases; as well as whether decaying learned Q-values toward the initial Q-values between the two phases in proportion to a decay parameter could account for forgetting between the two phases (indicated by x). Last, we tested whether fixing learning rate to 0 in the recall phase would fit better. We used WAIC (Vehtari et al., 2017) for model comparison. We found that the best fitting model included a separate learning rate and softmax inverse temperature for both phases (aabb; displayed in bold text).
